# Resting-State Connectivity of Auditory and Reward Systems in Alzheimer’s Disease and Mild Cognitive Impairment

**DOI:** 10.3389/fnhum.2020.00280

**Published:** 2020-07-17

**Authors:** Diana Wang, Alexander Belden, Suzanne B. Hanser, Maiya R. Geddes, Psyche Loui

**Affiliations:** ^1^Harvard College, Harvard University, Cambridge, MA, United States; ^2^Music, Imaging, and Neural Dynamics Laboratory (MIND), Northeastern University, Boston, MA, United States; ^3^Berklee College of Music, Boston, MA, United States; ^4^Brigham and Women’s Hospital, Harvard Medical School, Boston, MA, United States; ^5^Department of Music, Northeastern University, Boston, MA, United States

**Keywords:** resting-state fMRI, auditory, reward, dementia, Alzheimer’s disease, mild cognitive impairment

## Abstract

Music-based interventions (MBI) have become increasingly widely adopted for dementia and related disorders. Previous research shows that music engages reward-related regions through functional connectivity with the auditory system, but evidence for the effectiveness of MBI is mixed in older adults with mild cognitive impairment (MCI) and Alzheimer’s disease (AD). This underscores the need for a unified mechanistic understanding to motivate MBIs. The main objective of the present study is to characterize the intrinsic connectivity of the auditory and reward systems in healthy aging individuals with MCI, and those with AD. Using resting-state fMRI data from the Alzheimer’s Database Neuroimaging Initiative, we tested resting-state functional connectivity within and between auditory and reward systems in older adults with MCI, AD, and age-matched healthy controls (*N* = 105). Seed-based correlations were assessed from regions of interest (ROIs) in the auditory network (i.e., anterior superior temporal gyrus, posterior superior temporal gyrus, Heschl’s Gyrus), and the reward network (i.e., nucleus accumbens, caudate, putamen, and orbitofrontal cortex). AD individuals were lower in both within-network and between-network functional connectivity in the auditory network and reward networks compared to MCI and controls. Furthermore, graph theory analyses showed that the MCI group had higher clustering and local efficiency than both AD and control groups, whereas AD individuals had lower betweenness centrality than MCI and control groups. Together, the auditory and reward systems show preserved within- and between-network connectivity in MCI individuals relative to AD. These results motivate future music-based interventions in individuals with MCI due to the preservation of functional connectivity within and between auditory and reward networks at that initial stage of neurodegeneration.

## Introduction

Alzheimer’s disease (AD) is a severe and rapidly increasing problem, with over 5 million Americans suffering from this illness. Individuals with AD manifest variable but significant impairments in multiple cognitive, functional, and behavioral domains, including changes in mood and anxiety as well as the loss of memory and executive functions, which together affect activities of daily living (Marshall et al., 2011). While AD affects 10% of adults over age 65, an additional 15–20% of people above age 65 have mild cognitive impairment (MCI). MCI is defined as a noticeable decrement in cognitive functioning that goes beyond normal changes seen in aging and may progress to dementia (Petersen et al., 2009). Individuals with Amnestic MCI are at the highest risk of developing AD (Petersen et al., 2009). Because of this increased risk, early intervention is most likely to affect the temporal cascade of subsequent effects that lead to dementia (Vega and Newhouse, 2014).

In recent years, music-based interventions (MBIs) have become increasingly adopted for patients with AD and related disorders. Several randomized controlled trials have shown positive results in the effect of receptive MBIs on alleviating symptoms of cognitive decline, especially in improving mood and reducing stress when listening to familiar music. However, findings to date have been mixed—partly because of variability between subjects, small sample size, and because of differences between intervention protocols across studies (Vink and Hanser, 2018). Part of the challenge in understanding MBIs in neurodegenerative disease is that we do not yet know the influence of cognitive decline on brain networks that are involved in music processing. Advancing this knowledge could help researchers target more precisely when and how to administer MBIs and music therapy.

To date, the best available evidence suggests that music listening may motivate behavior through interactions between brain networks necessary for auditory predictions (such as predictions for melody, harmony, and rhythm) and the brain’s reward system. The auditory system is organized in subdivisions and processing streams that include cortical as well as subcortical regions. Cortical regions include bilateral Heschl’s and superior temporal gyri, but also extend towards superior temporal sulci and middle temporal gyri (Kaas and Hackett, 2000). Lesions to the right auditory cortex, encroaching into the right Heschl’s gyrus, results in perceptual deficits in perceiving pitch, while left temporal-lobe damage affects behavioral tasks that involve fine-grained temporal discrimination (Zatorre et al., 2002). There is abundant evidence showing that listening to music that we enjoy engages the dopaminergic reward system, indicating that rewarding music has similar properties to other rewarding experiences such as monetary gain and social stimulation (Salimpoor et al., 2013; Ferreri et al., 2019; Gold et al., 2019). When listening to personally pleasurable music, task fMRI has shown that cortical structures in the superior temporal lobe, which constitute an auditory brain network, are correlated in activity with areas in the reward system centering around the ventral striatum (Salimpoor et al., 2013; Martínez-Molina et al., 2016; Gold et al., 2019). Findings from structural neuroimaging have linked white matter connectivity between auditory and reward-related areas, specifically the posterior superior temporal gyrus to the anterior insula and ventromedial prefrontal cortex (vmPFC), to individual differences in reward sensitivity to music (Sachs et al., 2016; Loui et al., 2017; Martínez-Molina et al., 2019). These findings suggest that there is a neuroanatomical network that is known to be involved in deriving rewards from music listening (Belfi and Loui, 2020). Altogether, these two networks are well associated with behavioral data supporting their roles in the emotional processing of music.

In contrast to the structural neuroimaging and task neuroimaging literature, less is known about the intrinsic functional connectivity of the auditory and reward systems, and even less is known about how these patterns of intrinsic functional connectivity may vary in different stages of neurodegeneration. In a landmark study, Jacobsen et al. (2015) compared the brain activity of young adults listening to familiar and unfamiliar music in functional Magnetic Resonance Imaging (fMRI) and found that a specific region within the anterior cingulate cortex (ACC) was more active when listening to familiar music, likely part of the auditory prediction network. The authors then analyzed PET data of essential AD biomarkers in a region of interest derived from musical memory findings which included the caudal ACC and ventral pre-supplementary motor area. They showed that this musical memory region was relatively spared in AD, with minimal cortical atrophy and minimal disruption of glucose metabolism. These findings support the potential efficacy of MBIs in engaging these relatively preserved brain regions in individuals with AD. Overall, these findings raise the intriguing possibility that music processing might engage brain networks that are relatively spared in neurodegeneration. However, the fMRI results from music listening in this study were obtained from a healthy group of young adults. Thus, results could be explained by intrinsic differences between the different age groups rather than by the specific effects of music *per se*.

Another study specifically conducted resting-state fMRI (rsfMRI) and task fMRI during music listening in the same group of AD patients. King et al. (2019) showed that after listening to familiar music, patients with AD had increased functional connectivity in multiple regions including the default mode network (DMN) as well as the auditory and reward networks. The DMN is a resting state network that is involved in autobiographical memory, mind-wandering, and stimulus-independent thought, and has become a subject of intense interest especially as its connectivity is disrupted in AD (Greicius et al., 2004). Listening to familiar music has been associated with increased connectivity within the DMN, suggesting that music may aid autobiographical memory, a hypothesized role of the DMN (Kay et al., 2012). In this regard, the DMN may also play a role in enhancing the effects of music-based interventions through the activations of autobiographical memories by music listening. While these results provide strong evidence for the use of familiar music in music-based interventions, it remains unclear to what extent these differences in brain connectivity relate to symptom severity and stage of illness in AD. Taken together, it is clear that understanding the intrinsic functional connectivity of the auditory and reward systems, their connectivity to other areas such as the DMN, and how they change in the aging brain and in different clinical stages of AD may shed light on how and why music listening could help dementia and promote healthy aging.

The study of intrinsic functional brain networks is aided by recent developments in open science and open data sharing initiatives. The Alzheimer’s Disease Neuroimaging Initiative (ADNI) is a multicenter project that shares neuroimaging data from patients with AD, patients with MCI, and older adult controls (Jack et al., 2008). Data from ADNI offer a starting point from which to investigate intrinsic functional networks at different stages of cognitive decline. The overarching goals of the ADNI study are: (1) to detect AD at the earliest possible stage (pre-dementia) and identify ways to track the disease’s progression with biomarkers; (2) to support advances in AD intervention, prevention, and treatment through the application of new diagnostic methods at the earliest possible stages (when intervention may be most effective); and (3) to continually administer ADNI’s innovative data-access policy, which provides all data without embargo to all scientists in the world.

Here we ask how the auditory and reward systems are intrinsically connected in the healthy older adult brain, and how these connectivity changes at different stages of neurodegeneration. We compare resting-state networks of three age-matched groups: AD patients, MCI patients, and healthy controls (CN). We identify networks of regions with known roles in auditory prediction and reward and use them as seed regions of interest (ROIs) to compare the three groups in seed-based connectivity across the brain, in whole-brain second-level contrasts to assess between-group differences in resting-state functional connectivity, and in ROI-to-ROI connectivity within and across brain networks. Finally, we apply measures from graph theory to describe the complex network properties of the auditory and reward systems and to see how these networks change in different stages of dementia. Although data on responsiveness to MBI are not available in the ADNI dataset, we hope that the results from our analyses will inform future MBIs by characterizing the requisite auditory and reward networks and their trajectory in neurodegenerative disease.

## Materials and Methods

### Sample

We used open-source data from ADNI (Jack et al., 2008). From the available data, we limited our sample to patients who had magnetization-prepared, rapid-acquisition, gradient echo (MPRAGE) and rsfMRI scans that were free of artifacts, both of which met the specific scan parameters listed in the Procedures: MRI Acquisition. This resulted in 105 older adults (ages 55–90) matched in age and gender that were selected from the ADNI study set. In the Control group (*N* = 47), ages ranged from 56 to 86, with 27 females; in the MCI group (*N* = 47), ages ranged from 56 to 88, with 27 females; and in the AD group (*N* = 11), ages ranged from 55 to 86, with three females. The smaller sample of AD patients is due to lower data quality because of movement or noise artifacts from the available data. For each individual, two types of data were extracted for use in data analysis: structural MRI (MPRAGE) and functional MRI (fMRI).

### Procedures

#### MRI Acquisition

High-resolution T1 and resting-state images were acquired in a 3T SIEMENS scanner at multiple locations in the United States and Canada. The anatomical images were acquired using a T1-weighted, 3D, MPRAGE volume acquisition with a voxel resolution of 0.8 × 0.8 × 0.8 mm^3^ (TR = 2.3 s, TE = 2.95 ms, flip angle = 9°, Matrix *X* = 240 pixels, Matrix *Y* = 256 pixels, Matrix *Z* = 176 pixels, Mfg Model = Prisma_fit, Pulse Sequence = GR/IR, Slice Thickness = 1.2 mm).

Resting-state MRI was acquired as 197 contiguous echo-planar imaging (EPI) functional volumes, totaling to 9.85 min of resting-state fMRI data acquired from each subject (TR = 3 s; TE = 30 ms; flip angle = 90°; acquisition voxel size = 3.4375 × 3.4375 × 3.4375 mm^3^). Participants kept their eyes open during resting-state data acquisition.

#### MRI Preprocessing

Structural and functional MRI preprocessing were carried out with the CONN Toolbox^1^ (Whitfield-Gabrieli and Nieto-Castanon, 2012). In order, this consisted of functional realignment and unwarp (subject motion estimation and correction); functional centering to (0, 0, 0) coordinates (translation); functional slice-timing correction; functional outlier detection [Artifact Detection and Removal Tool (ART)-based identification of outlier scans for scrubbing]; functional direct segmentation and normalization (simultaneous grey/white/cerebrospinal fluid segmentation and Montreal Neurological Institute normalization); functional smoothing (spatial convolution with 8 mm Gaussian kernel); structural center to (0, 0, 0) coordinates (translation); structural segmentation and normalization (simultaneous grey/white/CSF segmentation and MNI normalization). An interleaved slice order was used for Siemens scans, intermediate settings (97th percentiles in normative samples), a global-signal *z*-value threshold of 9, subject-motion mm threshold of 2, structural target resolution of 1 mm, functionals target resolution of 3.4375 mm, and a bounding box of (90 −126 −72; 90 90 108) mm. Denoising steps for functional connectivity analysis included corrections for confounding effects of white matter and cerebrospinal fluid (Behazdi et al., 2007), and bandpass filtering to 0.008–0.09 Hz, which are the default values in CONN (Whitfield-Gabrieli and Nieto-Castanon, 2012).

#### Regions of Interest (ROIs) Selection

When choosing the ROIs for seed-based connectivity measures, we chose ROIs from the CONN default atlas (Whitfield-Gabrieli and Nieto-Castanon, 2012) which contains 185 ROIs and 32 networks. We selected 18 ROIs as auditory cortex regions based on previous literature which included all ROIs in the superior, middle, and inferior temporal lobes (Kaas and Hackett, 2000; Rauschecker and Tian, 2000): right anterior Superior Temporal Gyrus (aSTGR), left anterior Superior Temporal Gyrus (pSTGR), right posterior Superior Temporal Gyrus (pSTGR), left posterior Superior Temporal Gyrus (pSTGL), right anterior Middle Temporal Gyrus (aMTGR), left anterior Middle Temporal Gyrus (aMTGL), right posterior Middle Temporal Gyrus (pMTGR), left posterior Middle Temporal Gyrus (pMTGL), right temporooccipital Middle Temporal Gyrus (toMTGR), left temporooccipital Middle Temporal Gyrus (toMTGL), right anterior Inferior Temporal Gyrus (aITGR), left anterior Inferior Temporal Gyrus (aITGL), right posterior Inferior Temporal Gyrus (pITGR), left posterior Inferior Temporal Gyrus (pITGL), right temporooccipital Inferior Temporal Gyrus (toITGR), left temporooccipital Inferior Temporal Gyrus (toITGL), right Heschl’s Gyrus (HGR), and left Heschl’s Gyrus (HGL).

Then, we selected 18 ROIs as valuation and reward-related regions based on the previous literature (Belfi and Loui, 2020): right Insular Cortex (InsulaR), left Insular Cortex (InsulaL), Anterior Cingulate Gyrus (AC), Posterior Cingulate Gyrus (PC), right Frontal Orbital Cortex (FOrbR), left Frontal Orbital Cortex (FOrbL), right Caudate (CaudateR), left Caudate (CaudateL), right Putamen (PutamenR), left Putamen (PutamenL), right Pallidum (PallidumR), left Pallidum (PallidumL), right Hippocampus (HippocampusR), left Hippocampus (HippocampusL), right Amygdala (AmygdalaR), left Amygdala (AmygdalaL), right Accumbens (AccumbensR), left Accumbens (AccumbensL).

**Figure 1:**
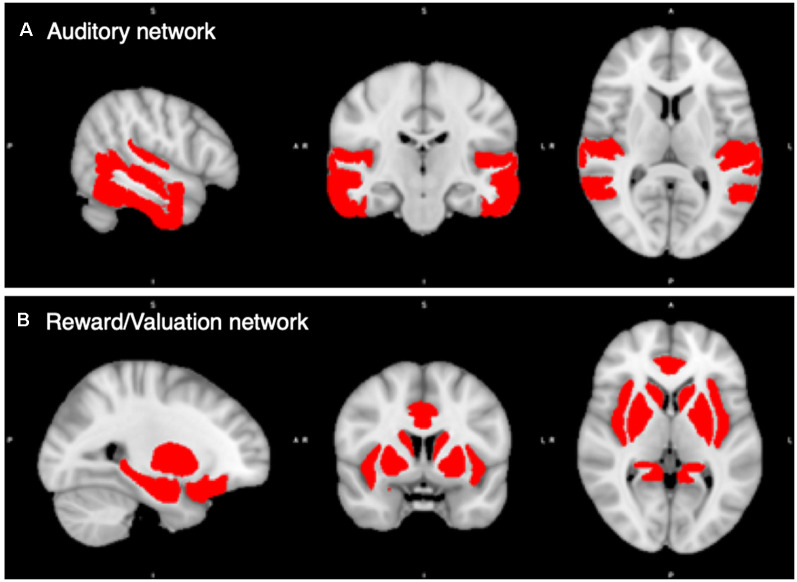
Regions of Interest (ROIs) in the auditory and reward/valuation networks from the CONN Toolbox. **(A)** Compilation of the 18 auditory ROIs from CONN. **(B)** Compilation of the 18 reward ROIs from CONN. See Supplementary Table S1 for a list of the ROIs used. The Auditory and Reward/Valuation networks in the figure become the two ROIs that we carry the rest of our analysis upon in this article.

Finally, we combined the 18 auditory ROIs into an *Auditory Network*, and the 18 rewards ROIs together into a Reward/Valuation Network (hereafter *Reward Network*). Figure 1 shows the auditory and reward network ROIs.

#### Seed-Based Connectivity Analyses

##### Within-Group Seed-Based Connectivity

Since we were interested in whole-brain connectivity patterns of the auditory and reward networks, we first seeded the auditory and reward networks defined above, and for each group of subjects, we extracted all voxels that were significantly functionally connected (using bivariate correlation) to the seed ROIs at the *p* < 0.05, Family Wise Error corrected level, to examine the connectivity patterns of each network in each group. Slices were chosen at the peak cluster for all three groups.

##### Between-Group Seed-Based Connectivity

Having identified seed-based connectivity patterns for each group, we then contrasted the three groups pairwise to test for between-group differences in seed-based connectivity from the auditory and reward networks for all pairs (i.e., CN > MCI, MCI > CN, CN > AD, AD > CN, MCI > AD, and AD > MCI). We used *p* < 0.05 family-wise error correction whenever possible. However, in contrasts where FWE correction did not show significant between-group differences in seed-based connectivity, we lowered the threshold to examine the contrasts at the less conservative *p* < 0.05 false discovery rate (FDR) cluster-size corrected level.

#### ROI-to-ROI Analyses

For pairwise correlations, ROI-to-ROI brain connectomes were created for all three groups that included all 36 ROIs. All significant positive *T*-values from the seed ROIs were extracted into 36 × 36 matrices.

#### Graph Theory Analyses

To compare the functional networks between groups, we utilized small-world brain networks which provide a useful approach to the investigation of functional connectivity (Bassett and Bullmore, 2006; Reijneveld et al., 2007; Hagmann et al., 2008; Ginestet and Simmons, 2011). Network analysis using graph theory measures yield powerful information about the community structure of brain regions in different groups of subjects, that cannot be accomplished using conventional measures of functional connectivity. We chose to focus on four graph parameters: clustering coefficients, strengths, betweenness centrality, and local efficiency. These parameters have been used to characterize brain networks and their degeneration in AD (Agosta et al., 2013; Brier et al., 2014; Khazaee et al., 2015). Furthermore, previous studies in music cognition have found these network statistics to be sensitive to musical training and musical aptitude (Loui et al., 2012; Belden et al., 2020). The *clustering coefficient* is a measure of functional segregation, indicating the fraction of neighboring nodes of each node that are also neighbors of each other, i.e., the cliquishness of a node (Watts and Strogatz, 1998). *Strength* is the sum of weights of links connected to each node (Latora and Marchiori, 2001). *Betweenness centrality* is a measure of centrality and denotes the number of shortest paths that pass through a given node (Hagmann et al., 2008). Finally, *local efficiency* is another measure of segregation; it is the inverse of the average shortest distance between each node in a subgraph and reveals the efficiency of each node within the network in transporting information (Ajilore et al., 2014).

Pairwise correlation coefficients (*r* values) for each of the 36 ROIs from the CONN atlas were extracted for every participant and averaged across each group to compute pairwise correlations and graph theory analyses. First, pairwise correlation matrices were extracted for all 36 ROIs from the CONN atlas, resulting in a 36 × 36 matrix for each participant in each group. These matrices were then analyzed using the Brain Connectivity Toolbox in MATLAB (Rubinov and Sporns, 2010). For each group, a series of proportional thresholds were tested, ranging from 5% to 100% of the overall connections. At each threshold level, the four network statistics were computed for each ROI and then averaged across participants for each group. We show graph theory statistics from thresholds ranging from 0.05 to 1.0 to visually show how group differences persist across a range of correlation thresholds. To avoid issues with multiple comparisons from performing tests at every threshold, we chose a proportional correlation threshold of 45% of the strongest connections for statistical analysis, as this captured a representative pattern of graph theory metrics for each group. To confirm that the variance of graph theory metrics was similar across the three groups (despite the smaller sample size in the AD group), two-sample Kolmogorov–Smirnov tests were used to compare the distributions of graph theory metrics between groups. All Kolmogorov–Smirnov tests were not significant (all *p* > 0.2); thus the network statistics did not appear to be differently distributed between groups. These group averages were then compared between groups using one-way ANOVAs to determine group differences in each network measure while correcting for a false-discovery rate of 0.05 for comparisons across the four network measures (Benjamini and Hochberg, 1995).

## Results

### Seed-Based Connectivity Analyses

#### Within-Group Seed-Based Connectivity

**Figure 2:**
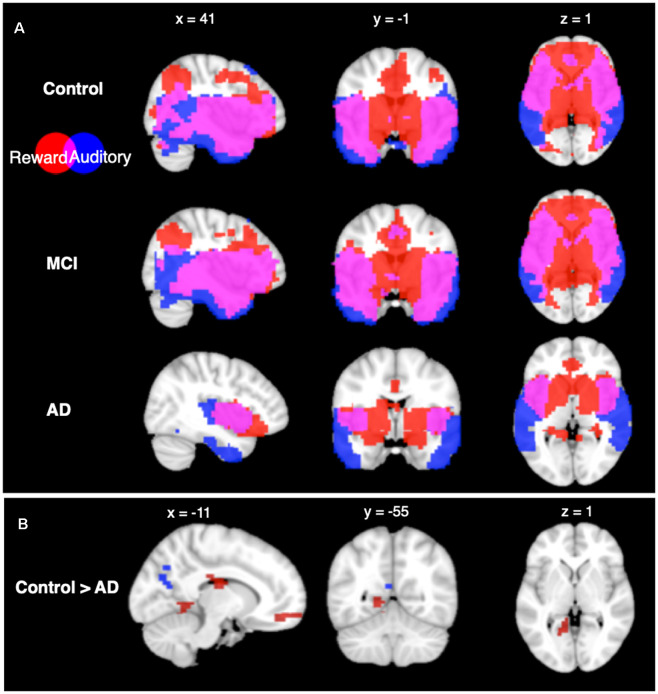
Seed based connectivity analysis. **(A)** Connectivity profiles of Control group (top row), mild cognitive impairment (MCI) group (middle row), and Alzheimer’s disease (AD) group (bottom row) for the auditory (blue) and reward (red) networks seed regions (*p* < 0.05, voxel-wise FWE corrected). **(B)** Connectivity profile differences comparing Control and AD groups seeded from auditory (blue) and reward (red) networks [*p* < 0.05, false discovery rate (FDR) cluster-size corrected].

Seed-based connectivity patterns from the auditory network for each group are shown in Figure 2. All groups showed highly significant auditory network functional connectivity to the auditory areas, including the STG, MTG, and ITG, at the *p* < 0.05 FWE-corrected level. The control and MCI groups additionally showed significant functional connectivity in the parietal, occipital, and frontal lobes. The AD group showed less significant functional connectivity than the other two groups, with the significant functional connectivity only observed in the temporal lobe, and not in the other lobes.

Seed-based connectivity from the reward network showed significant functional connectivity within areas of the reward network in all groups at the *p* < 0.05 FWE-corrected level. CN and MCI groups both have significant functional connectivity to the auditory network ROIs including the MTG and ITG, as well as significant overlap in areas that are functionally connected to auditory and reward ROIs in the frontal, parietal, and occipital lobes. In contrast, the AD group did not show connectivity in lateral frontal, parietal, or occipital lobes from the reward network ROIs.

#### Between-Group Seed-Based Connectivity

From the auditory network seed, between-group comparisons showed higher functional connectivity in the CN group compared to the AD group (*p* < 0.05 FDR cluster-size corrected) in the precuneus. From the reward network seed, between-group comparisons showed higher functional connectivity in the CN group compared to the AD group at the *p* < 0.05 FDR cluster-size corrected level in six regions: the cingulate cortex, the medial prefrontal cortex, the left lingual gyrus, the bilateral fusiform gyri, and superior parietal lobule. No other between-group differences were significant in seed-based connectivity.

### ROI-to-ROI Analyses

**Figure 3:**
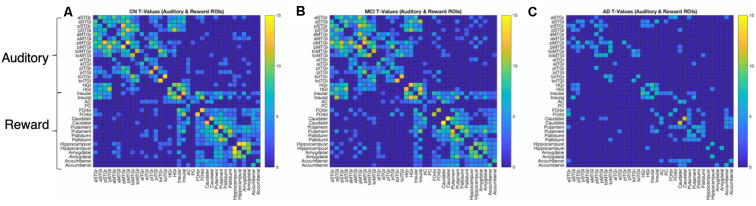
ROI-to-ROI connection matrices and corresponding brain connectomes. **(A)** Control group, **(B)** MCI group, **(C)** AD group showing significant positive correlations (*p* < 0.05, FDR corrected) between the auditory and reward regions. The colors correspond to the strength of the correlation between the two ROIs.

We further characterized within- and between-network connectivity across the 36 ROIs from the auditory and reward networks. Figure 3 shows the positive *T*-values of bivariate correlations between each pair of ROIs in each group. All three groups show higher connectivity within each network (auditory-auditory, reward-reward) than between networks (auditory-reward), as shown by higher *T* values within the diagonal quadrants (which represent auditory-auditory and reward-reward connectivity) than in the off-diagonal quadrants (which represent auditory-reward connectivity). The *T*-values are generally similar between CN and MCI groups. In contrast, the AD group has lower network connectivity overall.

### Graph Theory Analyses

**Figure 4:**
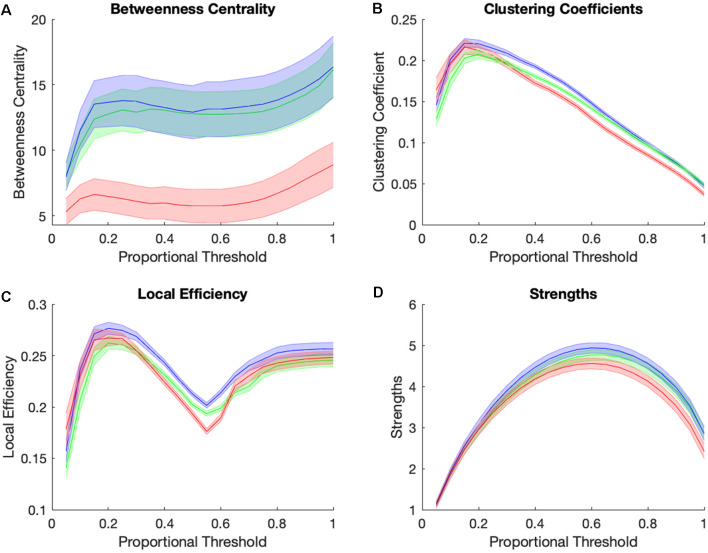
Group differences in small-world brain connectivity. Network measures of betweenness centrality **(A)**, clustering coefficients **(B)**, local efficiency **(C)**, and strengths **(D)** for Control group (green), MCI group (blue), and AD group (red) across a range of proportional thresholds (solid line = mean of all subjects’ ROIs for each group, error bar = standard error for all 36 CONN ROI’s averaged across subjects for each group).

Figure 4 shows graph theory measures for the three groups across a range of proportional thresholds. The main effects of the group were observed at a proportional threshold of 0.45 for betweenness centrality, clustering coefficient, and local efficiency, but not for strengths. Betweenness centrality showed significant group differences (*F*_(2,105)_ = 6.01, *p* = 0.0045, Benjamini-Hochberg corrected, Figure 4A), with the AD group showing significantly lower betweenness centrality while CN and MCI individuals did not differ. There was also a main effect of group for clustering coefficient (*F*_(2,105)_ = 15.08, *p* = 0.00000175, Benjamini-Hochberg corrected, Figure 4B) and for local efficiency (*F*_(2,105)_ = 11.57, *p* = 0.000028, Benjamini-Hochberg corrected, Figure 4C), with the MCI group showing highest clustering and local efficiency, followed by the CN and then AD group. Taken altogether, the MCI group is higher than the CN group in clustering and local efficiency and is similar to the CN group in betweenness centrality. The AD group is statistically indistinguishable from MCI and CN groups in strengths (Figure 4D) while being lower than others in clustering and local efficiency, and much lower than both other groups in betweenness centrality. In summary, the pattern of graph theory results show that MCI individuals are similar or even higher than CN individuals in clustering, local efficiency, and betweenness centrality, and have consistently high clustering and within the reward network relative to controls and AD individuals.

## Discussion

Although abundant research supports the interaction between auditory and reward systems in enabling pleasure in music listening, little is known about the intrinsic functional connectivity between the auditory and reward systems. Here, we defined an auditory network and a reward network based on previous studies and characterized their intrinsic functional connectivity using resting-state fMRI from a sample of AD, MCI, and age-matched controls. We found decreased functional connectivity within and between the two systems in AD individuals. These differences are observable in seed-based as well as ROI-to-ROI connectivity, and also in disruptions that affect clustering, local efficiency, and betweenness centrality of the overall network.

Importantly, we observe an overlap between seed-based connectivity patterns from the auditory network and the reward network. This overlap was observed in all three groups, centering around the anterior insula. Notably, there was no overlap among the ROIs chosen as the seed regions of the auditory and reward networks; thus the results are due to similar patterns in functional connectivity between the anterior insula and both the auditory and reward regions. The anterior insula is part of the salience network, which has been posited as a hub that enables alternating between default mode and executive control networks (Menon and Uddin, 2010). The present results extend that previous work by suggesting that the salience network, with the anterior insula at its core, may be key to interactions between large-scale brain systems more generally. This result has important implications. First, it supports the neuroanatomical model for the reward of music listening and music-based interventions, as laid out in Belfi and Loui (2020), which posits that the anterior insula is connected to both auditory and reward systems. This finding is also consistent with lesion mapping studies: cases of acquired musical anhedonia (i.e., the lack of emotional responses to music due to brain injury) mostly have lesions in the anterior insula (Griffiths et al., 2004; Satoh et al., 2011). Thus, the anterior insula seems to be a key region for deriving rewards from music listening.

The AD group showed less functional connectivity from the auditory network to the precuneus, and from the reward network to the cingulate cortex, the medial prefrontal cortex, the left lingual gyrus, the bilateral fusiform gyri, and superior parietal lobule. The precuneus is one of the most metabolically active areas in the brain (Cavanna and Trimble, 2006). The posterior precuneus (which is showing the difference in our study) is associated with episodic memory retrieval in fMRI studies (Cavanna and Trimble, 2006). In this context, the finding of lower auditory-seeded functional connectivity in the precuneus among AD individuals is consistent with less successful episodic memory retrieval in AD. The fact that this reduction is observed from auditory seeds suggests that the decrease in episodic memory retrieval may be specific to auditory access. On the other hand, findings in the precuneus may be more general, relating to the DMN which is disrupted in AD individuals (Greicius et al., 2004; Buckner et al., 2008).

Findings in the reward-seeded connectivity differences between the control group and AD group consisted of the cingulate cortex, the medial prefrontal cortex, the left lingual gyrus, the bilateral fusiform gyri, and superior parietal lobule. The medial prefrontal cortex, bilateral fusiform gyri, and lingual gyrus are also part of the DMN (Buckner et al., 2008; Christoff et al., 2016). The lingual gyrus is also coupled with the DMN as part of the overall brain system involved in mind-wandering or stimulus-independent thought (Christoff et al., 2016) and is also associated with better performance on creativity tasks (Belden et al., 2020). The superior parietal lobule, part of the dorsal attention network (Dixon et al., 2017), is related to memory, especially in music (Klostermann et al., 2009). Taken together, the regions that are under-connected to the reward network in the AD group are broadly consistent with brain networks associated with memory and stimulus-independent thought. These results are especially relevant in the present context as music-based interventions may draw upon both of these constructs (Hanser and Thompson, 1994).

Relative to AD individuals, MCI individuals show preserved functional connectivity, with no significant between-group differences in auditory-seeded or reward-seeded connectivity patterns from age-matched controls. Graph theory results showed higher degrees, strengths, clustering, and local efficiency in the MCI group than in both the AD and the control groups. Thus, the relationship between dementia severity and network connectedness appears to follow an inverse u-shaped curve, with the slightly impaired MCI group showing the strongest and most efficient connections across all the ROIs of the auditory and reward networks. This is different from previous findings in graph theory analysis of resting-state networks of MCI, AD, and CN groups (Seo et al., 2013). Using FDG-PET data, previous work has shown lower clustering in both MCI and AD groups compared to the CN group. However, those with very mild AD had lower clustering compared to those with mild AD (Seo et al., 2013). On the other hand, a more recent study found that the small world index, a summary network statistic, was significantly decreased in MCI converters who progressed to AD compared to stable MCI individuals who did not progress to AD (Miraglia et al., 2020). Taken together, the distinctions between MCI and AD may be more fine-grained than are captured in our study. Furthermore, as we were specifically interested in the auditory and reward networks we used only a subset of ROIs that represented these networks rather than ROIs covering the whole brain. Thus, our results should not be interpreted as generalizable towards the whole brain in all MCI individuals, but rather as results of a specific hypothesized network of regions important for deriving rewards from music listening.

In the present study, the finding of higher network statistics in auditory and reward network ROIs among MCI individuals may suggest that auditory and reward regions more readily connect in the MCI brain. This may have important implications for music therapy. As music-based interventions rely on the participants’ engagement with music and the activity and connectivity of the reward system are reflective of engagement in music and other domains (Kampe et al., 2001; Tamir and Mitchell, 2012; Salimpoor et al., 2013; Martínez-Molina et al., 2016; Ferreri et al., 2019), the current results may suggest that targeting individuals with MCI can capitalize on the heightened auditory-reward connectivity in MCI, thus offering the best chance for effective intervention.

AD individuals have less functional connectivity overall; however, this may be confounded by the fact that, due to limitations in data quality within the ADNI dataset, we had a smaller sample size of only 11 AD individuals, compared against *n* = 47 in control and MCI groups. Nevertheless, the AD group still shows some preserved overlap between auditory and reward systems in the anterior insula. This finding may also have implications for music-based interventions. Specifically, it may be possible to identify specific experiences that also engage the insula, and tailor music-based interventions to maximize these experiences. For example, the anterior insula has been implicated in specificity for voice processing and has been described as part of a voice-selective cortex (Abrams et al., 2013). Perhaps listening to music with the voice, or even engaging in vocalization in an active music-based intervention, maybe specific ways to tap into the reward system. Since the dopaminergic reward system is crucial for motivating behavior, understanding its connectivity patterns to the rest of the brain, and in different stages of the disease, offers insight into the design of effective interventions for diseases and disorders.

## Conclusion

We have identified an anatomical model of auditory and reward systems and characterized the functional connectivity within and between these systems in healthy older adults and older adults with MCI and AD. Results inform music-based interventions by highlighting the importance of focusing on the MCI population, as they have the most functional connectivity in their auditory and reward systems.

## Data Availability Statement

The datasets generated for this study are available on request to the corresponding author.

## Ethics Statement

Ethical review and approval was not required for the study on human participants in accordance with the local legislation and institutional requirements. The patients/participants provided their written informed consent to participate in this study.

## Author Contributions

PL conceptualized the idea behind this manuscript. DW acquired and preprocessed the behavioral and neuroimaging data, performed data analyses, and wrote the first draft. AB, SH, and MG provided feedback, guidance, and support on the conceptual and technical aspects of the study. All authors revised the manuscript and approved the submission.

## Conflict of Interest

The authors declare that the research was conducted in the absence of any commercial or financial relationships that could be construed as a potential conflict of interest.

## Footnotes

^1^http://www.nitrc.org/projects/conn

## Supplementary Material

TABLE S1 Auditory and reward brain regions and the XYZ-coordinates of their centers of gravity. The 36 ROI’s are from the default atlas in the CONN Toolbox (Whitfield-Gabrieli and Nieto-Castanon, 2012). Coordinates in millimeters in the Montreal Neurological Institute space.
