# Supplementary material for: Resting-State Connectivity of Auditory and Reward Systems in Alzheimer’s Disease and Mild Cognitive Impairment
Source: Front Hum Neurosci. 2020 Jul 17;14:280. doi: 10.3389/fnhum.2020.00280 (PMC7380265; doi:10.3389/fnhum.2020.00280)
Supplement: TABLE S1 — Auditory and reward brain regions and the XYZ-coordinates of their centers of gravity. The 36 ROI’s are from the default atlas in the CONN Toolbox (Whitfield-Gabrieli and Nieto-Castanon, 2012). Coordinates in millimeters in the Montreal Neurological Institute space. [file Data_Sheet_1.PDF]

Seed	Side	Coordinates		
		x	y	z
Auditory Network				
Anterior Superior Temporal Gyrus	Left	-56.17233662	-3.906445837	-7.970008953
	Right	57.50133452	-0.763345196	-10.16592527
Posterior Superior Temporal Gyrus	Left	-62.28850325	-29.1691974	3.79640533
	Right	61.34069304	-23.9858939	1.573443729
Anterior Middle Temporal Gyrus	Left	-57.46777317	-4.205255878	-22.13914246
	Right	57.88912197	-1.522025772	-24.50584357
Posterior Middle Temporal Gyrus	Left	-60.90641565	-27.35880095	-10.99670993
	Right	61.07506629	-22.52454969	-12.14985828
Temporooccipital Middle Temporal Gyrus	Left	-57.63985911	-52.99985324	0.8241855
	Right	58.18120733	-49.22239081	1.59748564
Anterior Inferior Temporal Gyrus	Left	-48.14201402	-4.974548137	-39.1910734
	Right	46.22887061	-2.40987285	-41.10583396
Posterior Inferior Temporal Gyrus	Left	-53.44317202	-28.4572097	-25.98756311
	Right	53.42195698	-23.46366737	-28.13352571
Temporooccipital Inferior Temporal Gyrus	Left	-51.81781305	-53.44356261	-16.53315697
	Right	54.14167857	-49.87783595	-16.73076313
Heschl's Gyrus	Left	-45.19664938	-20.3214998	7.192261667
	Right	46.11156095	-17.40440846	6.967611336
Reward Network				
Insular Cortex	Left	-36.3943	1.1868	0.0824
	Right	37.3847	2.5497	-0.1738
Anterior Cingulate Gyrus	Bilateral	0.80277298	18.29370562	24.34508732
Posterior Cingulate Gyrus	Bilateral	0.784845018	-36.62174953	29.97508841
Frontal Orbital Cortex	Left	-29.54284237	23.66228394	-16.57261043
	Right	29.11395129	23.07066013	-16.23143128
Caudate	Left	-12.78549849	8.976992796	9.737392517
	Right	13.30156062	10.01080432	10.49051621
Putamen	Left	-24.90149125	0.482649842	0.339546888
	Right	25.49574896	1.776008657	0.30344721
Pallidum	Left	-18.95779356	-5.120351024	-1.333890514
	Right	19.85048905	-4.005123428	-1.189101071
Hippocampus	Left	-25.17773788	-23.1916109	-13.80594092
	Right	26.49706667	-20.95893333	-14.25013333
Amygdala	Left	-22.99501151	-4.94666155	-17.73177283
	Right	23.08748616	-3.985234404	-17.68696936
Accumbens	Left	-9.463882619	11.496614	-7.170428894
	Right	9.368263473	12.20359281	-6.534431138
